# Wide bandgap BaSnO_3_ films with room temperature conductivity exceeding 10^4^ S cm^−1^

**DOI:** 10.1038/ncomms15167

**Published:** 2017-05-05

**Authors:** Abhinav Prakash, Peng Xu, Alireza Faghaninia, Sudhanshu Shukla, Joel W. Ager, Cynthia S. Lo, Bharat Jalan

**Affiliations:** 1 Chemical Engineering and Materials Science, University of Minnesota–Twin Cities, Minneapolis, Minnesota 55455, USA; 2 Department of Energy, Environmental, and Chemical Engineering, Washington University, St Louis, Missouri 63130, USA; 3 Materials Sciences Division, Lawrence Berkeley National Laboratory, Berkeley, California 94720, USA; 4 Energy Research Institute, Interdisciplinary Graduate School, School of Materials Science and Engineering, Nanyang Technological University, 50 Nanyang Avenue, Singapore 639798, Singapore; 5 Materials Science and Engineering, University of California at Berkeley, Berkeley, California 94720, USA

## Abstract

Wide bandgap perovskite oxides with high room temperature conductivities and structural compatibility with a diverse family of organic/inorganic perovskite materials are of significant interest as transparent conductors and as active components in power electronics. Such materials must also possess high room temperature mobility to minimize power consumption and to enable high-frequency applications. Here, we report n-type BaSnO_3_ films grown using hybrid molecular beam epitaxy with room temperature conductivity exceeding 10^4^ S cm^−1^. Significantly, these films show room temperature mobilities up to 120 cm^2^ V^−1^ s^−1^ even at carrier concentrations above 3 × 10^20^ cm^−3^ together with a wide bandgap (3 eV). We examine the mobility-limiting scattering mechanisms by calculating temperature-dependent mobility, and Seebeck coefficient using the Boltzmann transport framework and *ab-initio* calculations. These results place perovskite oxide semiconductors for the first time on par with the highly successful III–N system, thereby bringing all-transparent, high-power oxide electronics operating at room temperature a step closer to reality.

## Abstract (web summary)

With impressive electronic transport properties, wide bandgap perovskite oxides are promising transparent conductors. Prakash *et al*. report n-type BaSnO_3_ films with room temperature conductivity exceeding 10^4^ S cm^−1^ and investigate factors limiting carrier mobility.

Wide bandgap ternary oxides with perovskite structure have generated considerable excitement due to their impressive multi-functionality and the promising route they provide to potentially disruptive technologies for logic and resistive memory, utilizing both electronic and spintronic concepts^1^,^2^,^3^,^4^. Rapid progress with perovskite oxide heterostructures has also been made, including demonstration of strain-stabilized non-equilibrium electronic and magnetic properties, two-dimensional electron gas formation, quantum oscillation effects, and exotic magnetism and superconductivity^5^. However, the realization of this staggering range of functionalities at room temperature remains a grand challenge in the field. Carrier mobility in semiconducting perovskite oxides provides an ideal, and very technologically important, example^6^.

The best room temperature values of the mobility and conductivity in the model perovskite oxide semiconductor SrTiO_3_ have remained below 10 cm^2^ V^−1^ s^−1^ and 500 S cm^−1^, respectively for over 50 years^6^. Well-publicized advances with interfaces such as LaAlO_3_/SrTiO_3_ have similarly been restricted to low temperatures^5^. Very recently, bulk BaSnO_3_ (BSO) has shown significantly higher room temperature mobilities and corresponding conductivities. Luo *et al*.^7^, first discovered a room temperature mobility of 103 cm^2^ V^−1^ s^−1^ at a carrier concentration of 8 × 10^19^ cm^−3^ in bulk doped BSO crystals followed by the work of Kim *et al*.^8^,^9^ showing a much higher mobility of 320 cm^2^ V^−1^ s^−1^ and a conductivity value of 4 × 10^3^ S cm^−1^ at a carrier concentration of 8 × 10^19^ cm^−3^ (refs 8, 9). Owing to these behaviours, doped BSO has gained significant interest as a high-performance transparent conductor^7^,^8^,^9^,^10^,^11^,^12^. A favourable conduction band offset of BSO with structurally similar oxides such as SrTiO_3_ (STO) and LaAlO_3_ (LAO) further makes it a promising candidate for high mobility channel material in oxide heterostructures for both power electronic applications and fundamental physics study^6^,^13^,^14^,^15^.

However, thin films of BSO have shown significantly lower mobilities. The highest reported mobility in thin films grown using molecular beam epitaxy (MBE) is 150 cm^2^ V^−1^ s^−1^ on PrScO_3_ (110) at a carrier concentration of 7.2 × 10^19^ cm^−3^, decreasing to 80–120 cm^2^ V^−1^ s^−1^ for films on STO (001) between 6 × 10^19^–1 × 10^20^ cm^−3^, which results in an overall conductivity of 2 × 10^3^–3 × 10^3^ S cm^−1^ (ref. 16). The difference in mobility between bulk single crystals and thin films of BSO has been largely attributed to the substrate-induced misfit/threading dislocations^16^. Dislocations can introduce charged defects leading to compensation of carriers, and an overall reduced mobility. For instance, threading dislocation cores in GaN have been shown to consist of Ga-vacancy (*V*_Ga_) based defect complexes^17^, which form deep compensating acceptors^18^,^19^ together with space charge regions around the dislocation lines, resulting in enhanced electron scattering and thus reduced mobility^20^.

A similar effect can be expected from non-stoichiometry^21^, which also introduces charged point defects. Evidence that this effect is often at play in BSO is the observation of different mobility values for films grown via different synthesis routes despite having similar substrates and doping levels^7^,^9^,^22^,^23^. Even homoepitaxial films^24^ show much lower mobility compared to bulk single crystals suggesting an important role of point defects. Furthermore, films grown by MBE show a large variation in the mobility values despite having identical carrier concentration^16^. These results raise the question of how non-stoichiometry and dislocations in films influence electronic transport. It further begs the question as to what scattering mechanisms limit the electron mobility and therefore the conductivity in BSO films and what can be the ultimate mobility in BSO if defects are minimized. It is also noteworthy that there are no transport studies in BSO below a doping level of 10^19^ cm^−3^ raising further questions regarding the identity of the compensating defects.

To understand the role of dislocations, non-stoichiometry, and chemical dopants on electronic transport, and to examine mobility-limiting scattering mechanisms in doped BSO, we combine experiments and modelling using the Boltzmann transport and density functional theory (DFT) calculations. First, we investigate the influence of dislocations on the carrier concentration and mobility in doped BSO by tailoring the dislocation density by varying undoped buffer layer thickness. Second, we investigate the role of charge compensation on the critical density for the metal-to-insulator transition, carrier concentration, mobility and the Seebeck coefficient. Finally, we calculate the band structure, mobility and Seebeck coefficient to shed light on the scattering mechanisms limiting the mobility at different carrier concentrations and temperatures and provide the upper limits for mobility in a dislocation-free material.

## Results

### Thickness optimization of buffer and active layers

All BSO/STO heterostructures were prepared using hybrid MBE. Growth conditions were chosen to yield phase pure, epitaxial and stoichiometric films on STO substrates^21^,^25^. (See Supplementary Figs 1 and 2 for X-ray diffraction pattern and atomic force micrograph). The lattice mismatch of BSO is −5.12% (compressive) with the STO(001) substrate, resulting in the formation of misfit dislocations and strain relaxation for films with thickness above 1 nm (ref. 25). The density of misfit dislocations is largest near the film/substrate interface, which reduces the carrier mobility and also affects defect densities. Inserting a thick insulating (undoped) buffer layer between the substrate and the doped (active) layer may reduce the defect density in doped layers grown on top of it^26^. It must, however, be noted that dislocations remain present in doped layers as threading dislocations^25^, which are inevitable without a lattice-matched substrate.

**Figure 1:**
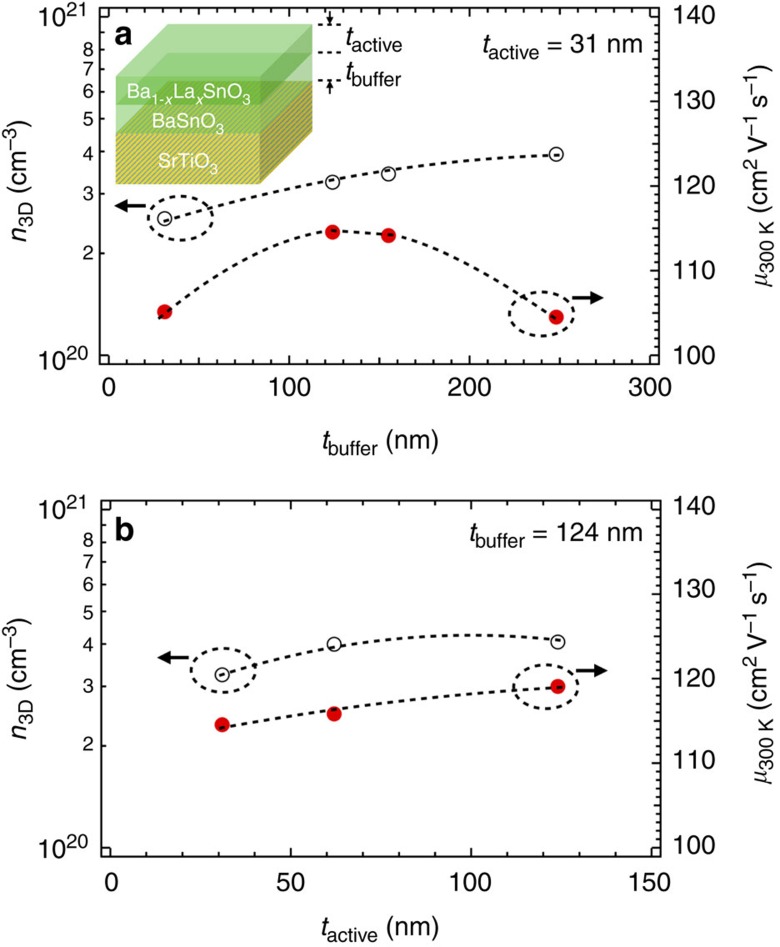
Effect of buffer and active layer thicknesses on room temperature carrier density and mobility. Variation of *n*_3D_ (black open circles) and *μ*_300 K_ (red solid circles) in doped BaSnO_3_ films as a function of (**a**) *t*_buffer_ for fixed *t*_active_=31 nm, and (**b**) *t*_active_ for fixed *t*_buffer_=124 nm. Inset shows a schematic of the structure.

To investigate the role of dislocations on the electronic transport, we grew a series of 31 nm La-doped BSO/*t*_buffer_ BSO/STO(001) films, as illustrated in the inset of Fig. 1a. Here, we fix the thickness of the active layer for which the transport will be evaluated while varying *t*_buffer_ between 30 and 250 nm. Dopant density in the active layer was kept constant by fixing the La-cell temperature (*T*_La_) at 1230 °C. Figure 1a shows room temperature carrier concentration (*n*_3D_) and mobility (*μ*_300 K_) as a function of *t*_buffer_. *n*_3D_ increases monotonically between 2.5 × 10^20^ cm^−3^ and 4 × 10^20^ cm^−3^ whereas *μ*_300 K_ first increases to 115 cm^2^ V^−1^ s^−1^ at *t*_buffer_=124 nm and then decreases. The fact that *n*_3D_ increases with increasing *t*_buffer_, which in turn decreases dislocation density in active layer (See Supplementary Fig. 3) suggests charge compensation being operative, and that threading dislocations act as acceptor-like defects. We interpret the decrease in *μ*_300 K_ for *t*_buffer_>124 nm is due to scattering from increased surface roughness (See Supplementary Fig. 4)^27^. Having determined the optimal buffer layer thickness which is similar to what has been reported by Park *et al*.^27^, we then grew a series of *t*_active_ La-doped BSO/124 nm BSO/STO(001) films keeping the dopant density (*T*_La_=1230 °C) fixed (Fig. 1b). No significant change in *n*_3D_ and *μ*_300 K_ was observed indicating no surface depletion effect or enhanced scattering at the buffer/active interface. That is, the active layer appears to be spatially uniform from a transport point of view. For rest of the study, we have chosen *t*_buffer_=124 nm and *t*_active_=124 nm unless otherwise stated.

### Metal-insulator transition and charge compensation in doped BaSnO3 films

**Figure 2:**
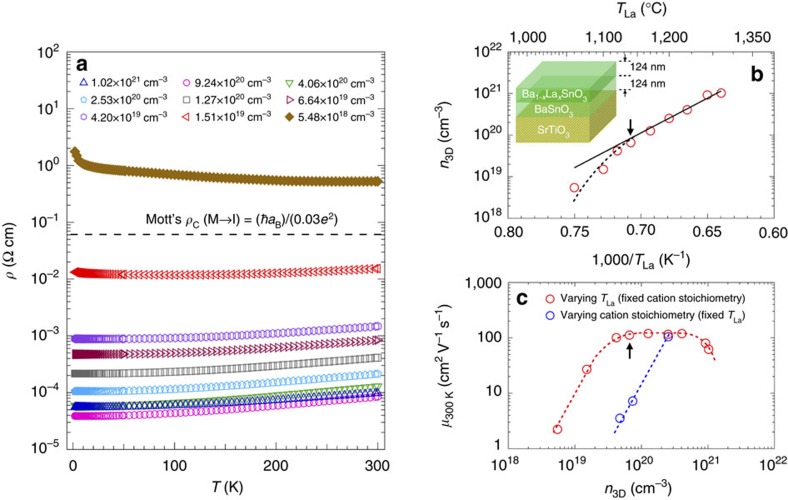
Temperature dependence of resistivity and the influence of charge compensation on room temperature carrier density and mobility. (**a**) Resistivity (*ρ*) of doped BaSnO_3_ films as a function of temperature (*T*) for different free carrier concentration measured at 300 K. (**b**) Free carrier concentration (*n*_3D_) at 300 K as a function of La cell temperature (*T*_La_) representing dopant concentration. Inset shows the schematic of the structure used, and (**c**) *μ*_300 K_ versus *n*_3D_ for stoichiometric (red open circles) and Ba-deficient films (blue open circles). Non-stoichiometric films had 31 nm of buffer and active layer thicknesses. Black arrows mark the onset of charge compensation.

To systematically investigate the effect of charge compensation due to dislocations on electronic transport, we prepared a doping series of 124 nm Ba_1−*x*_La_*x*_SnO_3_/124 nm BSO/STO(001) heterostructures. The value of *x*, and thereby *n*_3D_ was controlled by varying *T*_La_ at a fixed Ba/Sn beam equivalent pressure (BEP) ratio. The oxygen flux was also kept fixed. Figure 2a shows the resistivity (*ρ*) versus *T* as a function of *n*_3D_. Dashed line in Fig. 2a at 0.06 Ω-cm corresponds to the calculated values of *ρ* for metal-to-insulator transition based on Mott's minimum metallic conductivity (*σ*_min_) criterion for a degenerately doped semiconductor^28^:


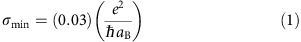


where *a*_B_ is the Bohr radius with its value between 2.5 × 10^−7^ cm and 5.0 × 10^−7^ cm for BSO assuming an effective mass (m*) of 0.2 *m*_e_–0.4 *m*_e_ (refs 22, 29), and a dielectric constant of 20 (ref. 30). A small upturn in *ρ*(*T*) was observed at low-temperatures and attributed to weakly localized transport. *ρ*(*T*) increases with decreasing *n*_3D_ leading to a metal-to-insulator transition between 5.48 × 10^18^ cm^−3^<*n*_3D_<1.51 × 10^19^ cm^−3^. The theoretical critical carrier density (*n*_C_) for the Mott metal-to-insulator transition in an uncompensated, degenerately doped semiconductor is shown in ref. 28:


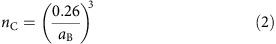


Using equation 2, we calculate *n*_C_ for metal-to-insulator transition between 1 × 10^17^ cm^−3^ to 1.0 × 10^18^ cm^−3^, which is an order of magnitude smaller than our experimental value. This result suggests the existence of charge compensation in agreement with the presence of dislocations, as also observed in doped compensated semiconductors^31^,^32^.

Next, we discuss the influence of charge compensation on *n*_3D_ and *μ*_300 K_. Figure 2b shows a semi-log plot of room temperature *n*_3D_ as a function of inverse of *T*_La_. It is noted that 1/*T*_La_ is directly related to the dopant density (*N*_dopant_) as 
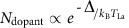
, where Δ and *k*_B_ are activation energy of evaporation of La, and the Boltzmann constant respectively. For low 1/*T*_La_ (high *N*_dopant_), *n*_3D_ first decreases linearly on a logarithmic scale, as one would expect with increasing 1/*T*_La_ if La is the source of electron and if it is fully-activated (See Supplementary Fig. 5). For high 1/*T*_La_ (low *N*_dopant_), *n*_3D_ however decreases faster, that is, deviates from linearity, indicating electrons are being trapped at the charged dislocations present in the film resulting in lower carrier concentration. This trend is in agreement with the charge compensation being operative below *n*_3D_≤6.64 × 10^19^ cm^−3^ (marked by an arrow) and is remarkably similar to the behaviour observed in doped III–N systems with dislocations^17^,^18^,^19^,^20^,^33^. Fig. 2c shows *μ*_300 K_ versus *n*_3D_ for two series of samples: doped Ba_1−*x*_La_*x*_SnO_3_ with a fixed cation stoichiometry but different *x* (red circle); and doped Ba_1−*x*−*y*_La_*x*_SnO_3_ with a fixed *x* but different *y*≥0 (blue circle). It is noted that *y*=0 corresponds to the cation stoichiometric composition with *n*_3D_=2.53 × 10^20^ and *μ*_300 K_=105 cm^2^ V^−1^ s^−1^. The value of *y*, and thereby Ba vacancies 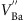 was controlled by varying the Ba/Sn BEP at a fixed Ba BEP. We first discuss the stoichiometric samples (red circle) with different *n*_3D_. We observe with decreasing carrier density, 4 × 10^20^ cm^−3^<*n*_3D_<1 × 10^21^ cm^−3^, mobility first increases; then remain unchanged between 6.64 × 10^19^ and 4.06 × 10^20^ cm^−3^ followed by a steeper decrease at *n*_3D_≤6.64 × 10^19^ cm^−3^ (marked by an arrow). Remarkably, the carrier density at which *μ*_300 K_ begins to decrease is identical to the density at which non-linearity occurs and the compensation kicks-in, as illustrated in Fig. 2b. This result suggests the decrease in mobility for low *n*_3D_ is due to scattering and compensation from charged defects largely owing to dislocations in the film, and is remarkably similar to the prior results obtained from GaN thin films^19^.

To further reveal scattering due to charged defects in low doping regime, we now turn to the second series of samples, Ba_1−*x*−*y*_La_*x*_SnO_3_ where *x* was kept constant, and *y* (thereby 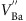) was varied (blue circle in Fig. 2c). We observe a reduction of *n*_3D_ accompanied by a decrease in mobility with increasing 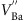, in agreement with the theoretical prediction of Ba-vacancies being an acceptor-like defect^34^. Most importantly, we observe that the functional dependence of *μ*_300 K_ versus *n*_3D_ is similar for both sets of samples in the low doping regime. Besides indicating a similar scattering potential around dislocation cores and Ba-vacancies, this result also suggests that the dislocation cores in nominally stoichiometric films may consist of Ba-vacancies. Future experimental and theoretical studies should clearly focus on studying the composition of dislocation cores and electrostatic scattering potentials around them.

### Mobility-limiting scattering mechanisms

**Figure 3:**
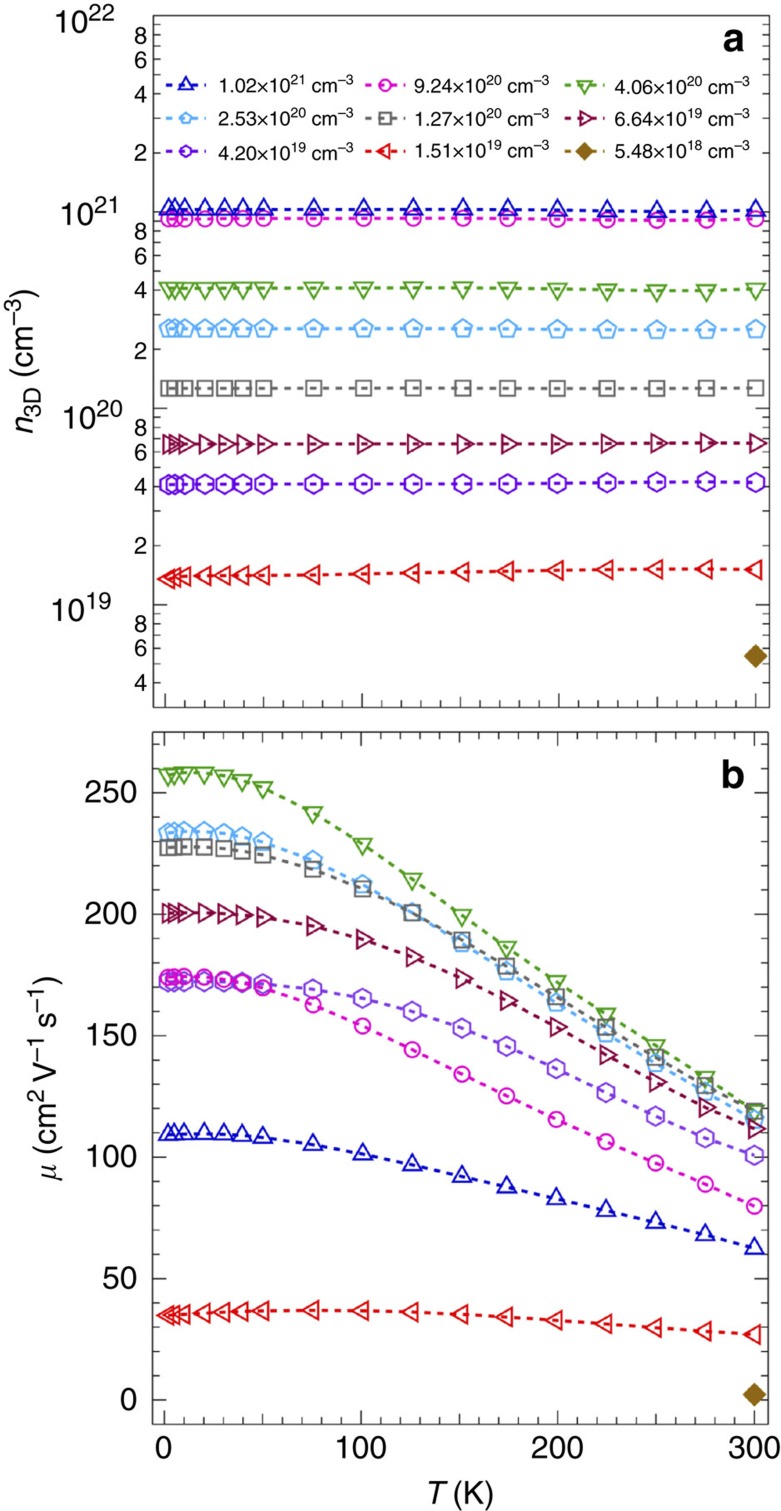
Temperature dependence of carrier concentration and mobility. (**a**) *n*_3D_, and (**b**) *μ* as a function of temperature (*T*) for doped BSO films with different *n*_3D_.

To get more insight into the transport in BSO films, we measured the temperature dependence of *n*_3D_ and *μ* for different dopant concentrations, as shown in Fig. 3. No carrier freeze-out was found down to 1.8 K for all measured electron concentrations (Fig. 3a), indicating that La-doped BSO forms a degenerate semiconductor. No T-dependence of the sample with electron concentration of 5.48 × 10^18^ cm^−3^ is shown due to the non-ohmic contacts at *T*<300 K. A strong *T*-dependence of *μ* was observed for all films (Fig. 3b) indicating different scattering mechanisms at play.

To examine the role of individual carrier scattering mechanisms, we calculated *μ* as a function of *T*. For this purpose, we used AMSET: *ab initio* model for calculating the mobility and Seebeck coefficient using the Boltzmann transport equation (BTE)^35^,^36^. AMSET has proven reliable for the calculation of mobility and Seebeck coefficient of several other semiconductors, including ZnS (ref. 37), GaAs and InN (ref. 35), whose transport properties are also governed by a single conduction band, like BSO. The model setup and parameters used are summarized in the method section, with additional details found in the literature^35^. In this model, the DFT band structure is used to calculate the group velocities of the carriers, which in turn are used to compute various carrier-scattering rates that enable us to explicitly solve the BTE; we then calculate the perturbation to the electronic distribution in the presence of low-electric field or thermal gradient, which gives us the mobility or Seebeck coefficient, respectively, of BSO. We considered both inelastic scattering (longitudinal polar optical phonon, (LO)) and elastic scattering (ionized impurity (IMP), acoustic phonon deformation potential (AC), transverse optical phonon (TO), piezoelectric (PE) and charged dislocation (DIS)) mechanisms. In our model, charged threading dislocations are treated as acceptor-like (negative charge) defects. We note that it is a reasonable assumption owing to its influence on the carrier density^38^. Moreover, it is also natural to think that in the presence of charged dislocations (negative charge), ionized dopants (positive charge) and electronic carrier (negative charge) in the doped BSO film, there must also exist other types of donor-like (positive charge) defects to maintain charge neutrality. We therefore can write a charge neutrality condition^35^:


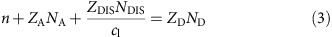


where *Z* represents the charge; A stands for acceptor (− charge) and D for donor (+ charge); *c*_l_ is the lattice constant of BaSnO_3_. Note the donor (*N*_D_) here represents both charged dopants and other donor-like defects to compensate for charged dislocations. For n-doped BSO films, *N*_D_≫*N*_A_ and thus acceptor term *Z*_A_*N*_A_ can be neglected. Using the formulation from refs 35, 39, we define an overall ionized impurity concentration, *N*_IMP_ in our films as (assuming *N*_A_=0 as discussed above):


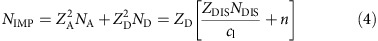


where *n* is the electron concentration of the samples and *Z*_D_ is the charge state of the donor. Equation 4 shows how *N*_IMP_ depends not only on the electron concentration, *n*, but also on the concentration of charged threading dislocations, *N*_DIS_. As seen in equation 5 (in method section), IMP scattering rate is proportional to *N*_IMP_.

**Figure 4:**
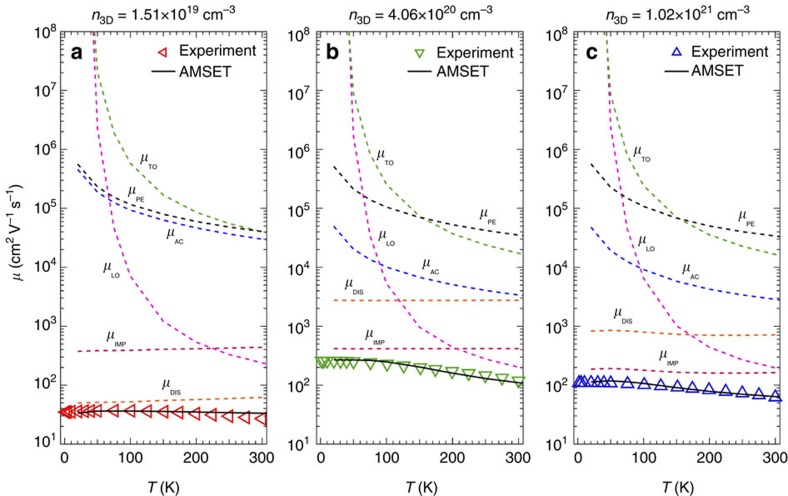
Temperature dependence of mobility and individual contributions to the mobility from different scattering mechanisms. *μ* versus *T* for doped BaSnO_3_ films, (**a**) *n*_3D_=1.51 × 10^19^ cm^−3^, (**b**) *n*_3D_=4.06 × 10^20^ cm^−3^, and (**c**) *n*_3D_=1.02 × 10^21^ cm^−3^. Contributions from different carrier scattering mechanisms are shown with dotted curves. Dislocation density as calculated from the model were—(**a**) *Z*_D_*Z*_DIS_*N*_DIS_=2.3 × 10^12^ cm^−2^, (**b**) *Z*_D_*Z*_DIS_*N*_DIS_=7.0 × 10^11^ cm^−2^, and (**c**) *Z*_D_*Z*_DIS_*N*_DIS_=2.8 × 10^12^ cm^−2^ respectively for the three samples, where *Z*_D_ and *Z*_DIS_ are the overall charge states of the donors and charged dislocations respectively. See method section for more details.

Figure 4 shows experimental (symbols) and calculated values (solid line) of *μ* versus *T* for three representative samples with *n*_3D_ belonging to low, intermediate and high density regimes of Fig. 2c. We also show individual contributions to the overall mobility due to different scattering mechanisms (dashed lines). We only show calculated mobility down to 20 K because below this temperature, the Fermi–Dirac distribution becomes step-like and the numerical integration of the distribution and density of state to calculate *n*_3D_ becomes unreliable. As *N*_DIS_, *Z*_D_ and *Z*_DIS_ are not known for these samples, we use them as a fitting parameter for calculating *μ*, which yields *Z*_D_*Z*_DIS_*N*_DIS_ between 7 × 10^11^ and 3 × 10^12^ cm^−2^. Ionized impurities and dislocations both are charged defects; the overall functional dependence of *μ* on *T* thus remains same for the two scattering mechanisms. LO phonon scattering has a weak dependence on *n*_3D_, and therefore remains nearly same for all three films. AC scattering, TO phonon scattering, and PE scattering, on the other hand, are dependent on *n*_3D_. However, their dependence on *n*_3D_ becomes weaker at higher carrier concentrations.

Looking at the dominant scattering processes for films with low doping (*n*_3D_=1.51 × 10^19^ cm^−3^) and high doping (*n*_3D_=1.02 × 10^21^ cm^−3^), dislocation scattering and ionized impurity scattering limit the mobility respectively at all temperatures (Fig. 4a,c). However, for films with intermediate doping (*n*_3D_=4.06 × 10^20^ cm^−3^), mobility is mostly limited by ionized impurity scattering below 200 K (Fig. 4b), while at higher temperatures mobility is governed by LO phonon scattering. Moreover, from Fig. 4a–c, dependence of *μ*_IMP_ on the concentration decreases for *n*_3D_=1.02 × 10^21^ cm^−3^, whereas it remained nearly unchanged between *n*_3D_=1.51 × 10^19^ cm^−3^ and *n*_3D_=4.06 × 10^20^ cm^−3^. This seems counter intuitive as one would naively think IMP scattering should increase with increasing *n* and therefore *μ*_IMP_ should decrease proportionately. We interpret this behaviour owing to a combined effect of *N*_IMP_ and the charge screening on IMP scattering with different doping density. For instance, using equation 4, the values of *N*_IMP_ are 7 × 10^19^ cm^−3^ and 4.33 × 10^20^ cm^−3^ for samples presented in Fig. 4a,b respectively (assuming *Z*_D_=1) suggesting higher *μ*_IMP_ in Fig. 4a if *N*_IMP_ is the only mobility-limiting factor. However on the other hand, the calculated inverse charge screening lengths, *β* for samples presented in Fig. 4a–c are about 0.57 nm^−1^, 0.98 nm^−1^, 0.98 nm^−1^ respectively at 300 K implying increased IMP scattering rates (See method section) and lower *μ*_IMP_ for the sample in Fig. 4a. Using the same argument, lower *μ*_IMP_ in Fig. 4c can also be understood owing to increased *N*_IMP_. See the method section for more information on charge screening.

Figure 4a–c further revealed *n*-dependence of *μ*_DIS_ increases from *n*_3D_=1.51 × 10^19^ cm^−3^ to 4.06 × 10^20^ cm^−3^ and then decreases for *n*_3D_=1.02 × 10^21^ cm^−3^. Using *β*, we calculate the DIS scattering rate, which is larger for sample with *n*_3D_=1.51 × 10^19^ cm^−3^; and almost similar for other two samples. In addition, we found *Z*_D_*Z*_DIS_*N*_DIS_ of 2.3 × 10^12^, 7.0 × 10^11^ and 2.8 × 10^12^ cm^−2^ for samples in Fig. 4a–c, respectively. Combining the effect of *N*_DIS_ and the charge screening on DIS scattering, *μ*_DIS_ is thus expected to possess the lowest value for sample with *n*_3D_=1.51 × 10^19^ cm^−3^, increasing for *n*_3D_=1.02 × 10^21^ cm^−3^ and being largest for *n*_3D_=4.06 × 10^20^ cm^−3^, as illustrated in Fig. 4.

**Figure 5:**
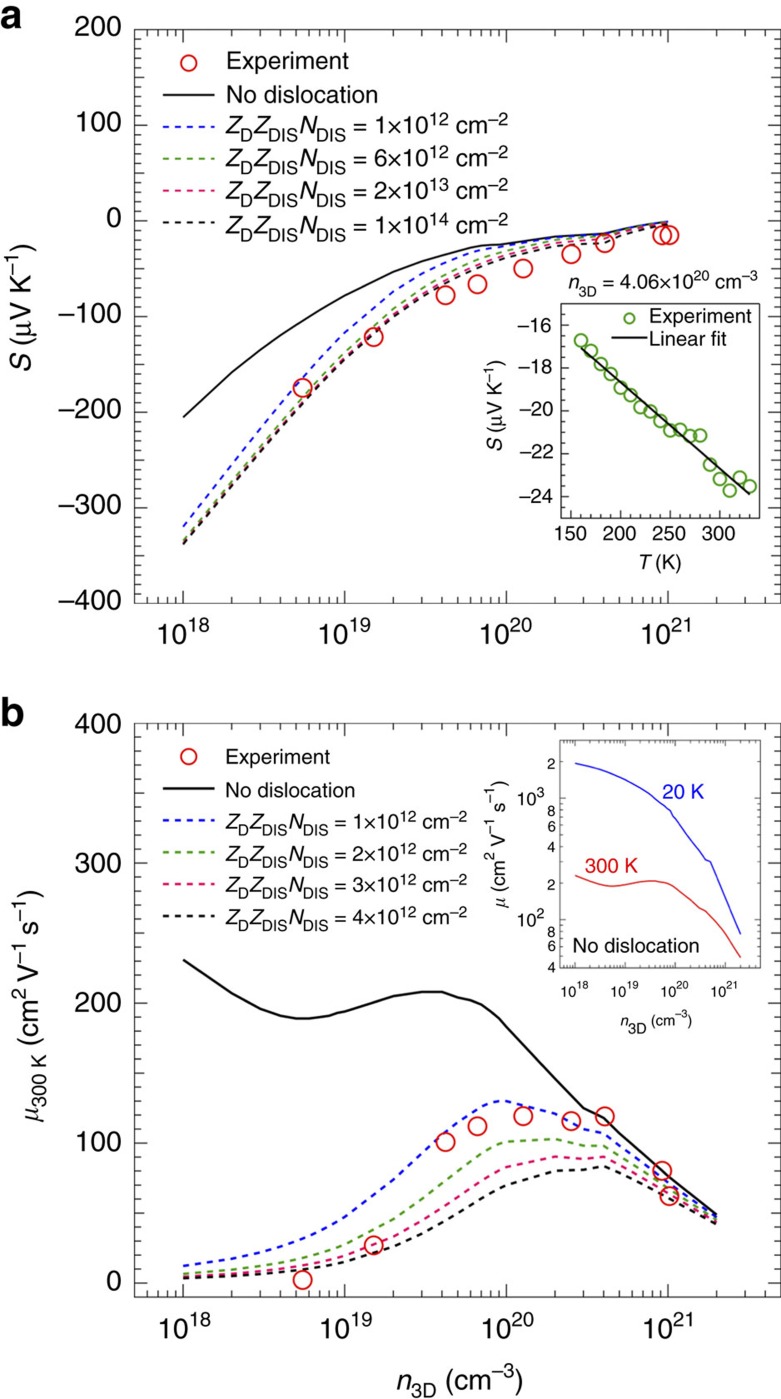
Comparison of experimental and calculated values of Seebeck coefficient and mobility as a function of carrier concentration with and without dislocations. (**a**) Seebeck coefficients (*S*) of doped BaSnO_3_ films at 300K as a function of *n*_3D_. Experimental data are shown as red circles and calculated values are represented using solid line for no dislocation, and using dotted lines for different *Z*_D_*Z*_DIS_*N*_DIS_. Inset shows the *T*-dependent *S* for *n*_3D_=4.06 × 10^20^ cm^−3^, consistent with n-type conduction. (**b**) Room-temperature mobility (*μ*_300 K_) as a function of *n*_3D_. Experimental data are shown in red circles while calculated values are represented with solid (without dislocation) and dotted lines (with varying dislocation density). Inset shows the variation of *μ* at two different temperatures (300 K and 20 K) for films with no dislocation.

As a further check of our model, we perform measurements and calculations of the Seebeck coefficients (*S*) as a function of *n*_3D_ (Fig. 5a). Inset shows *T*-dependent Seebeck coefficient for a representative sample with *n*_3D_=4.06 × 10^20^ cm^−3^, indicating negative *S* consistent with n-type carriers. Calculations of *S* were performed accounting for all the scattering mechanisms as described above for calculating *μ*, with and without *N*_DIS_. Significantly, overall trend of experimental value of *S* versus *n*_3D_ is similar to the calculated values with dislocations, in agreement with our transport results. The calculation further reveals that the Seebeck coefficient is independent of *N*_DIS_ in the high doping regime whereas increasing *N*_DIS_ seems to enhance and then saturate the Seebeck coefficient in the low doping regime.

To further examine the self-consistency of AMSET model, we plot in Fig. 5b the calculated dependence of *μ*_300 K_ versus *n*_3D_ for different *N*_DIS_. *μ*_300 K_ is also calculated for the case, when no dislocations are present. We overlay experimental values of *μ*_300 K_ (red circles) on the same plot for comparison. The calculated values are again in good agreement with the experiment. The result suggests that our films may have different *N*_DIS_ despite of identical *t*_buffer_, which may be due to different doping levels. We note that the calculated mobilities using AMSET are sometimes underestimated as the record room temperature mobility in bulk BSO has been 320 cm^2^ V^−1^ s^−1^ at *n*_3D_=8 × 10^19^ cm^−3^, whereas the calculated value in Fig. 5b is 195 cm^2^ V^−1^ s^−1^ at a similar doping level, so these values should be treated as lower bounds. Most importantly, in addition to providing numerous insights into electronic transport behaviour of BaSnO_3_, our calculations also suggest directions for future experiments. For instance, calculations reveal the overall trend of *μ*_300 K_ versus *n*_3D_ if there were no dislocations in the film. It tells that with decreasing *n*_3D_, *μ*_300 K_ with no dislocation will increase owing to reduced impurity scattering. For 5 × 10^18^ cm^−3^<*n*_3D_<4 × 10^19^ cm^−3^, *μ*_300 K_ is expected to decrease due to stronger interaction between low density electrons (that is, lower Fermi level) and optical phonons until electron-phonon interactions become much weaker (that is, reduced LO phonon scattering) resulting again in an increase of *μ*_300 K_ for *n*_3D_<5 × 10^18^ cm^−3^. In contrast, mobility at low temperature (20 K) is expected to show a monotonically increasing behaviour with decreasing *n*_3D_, reaching towards a value of 2,000 cm^2^ V^−1^ s^−1^.

### New outlook and opportunities

**Figure 6:**
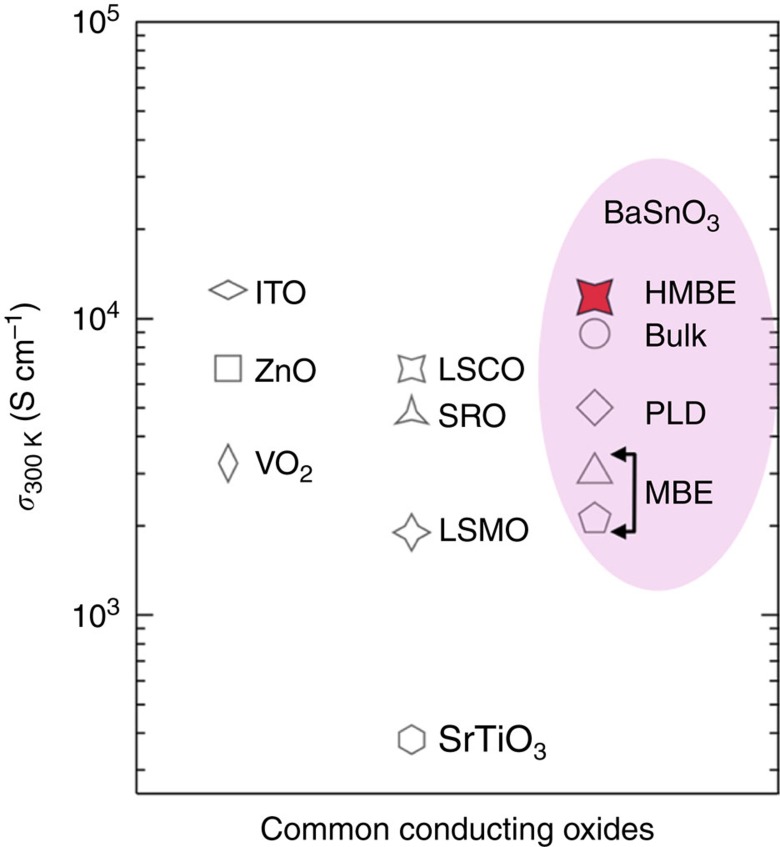
Comparison of conductivity values for common conducting oxides. Compilation of highest room temperature conductivity *σ*_300 K_ of BaSnO_3_ bulk single crystal and films grown by various deposition techniques (taken from refs 9, 16, 22) including hybrid molecular beam epitaxy. The highest known *σ*_300 K_ for common transparent conducting oxides (ITO=indium tin oxide, ZnO) and other metallic oxides as conductive electrodes for high frequency applications (VO_2_, LSCO=La_0.5_Sr_0.5_CoO_3_, SrTiO_3_, LSMO=La_0.7_Sr_0.3_MnO_3_, SRO=SrRuO_3_) are shown for comparison (refs 49, 50, 51, 52, 53, 54, 55).

To benchmark our films with respect to the best wide gap oxide conductors, we show in Fig. 6 the highest reported value of room temperature conductivity *σ* (300 K) for different oxides, which are often used as metallic electrodes and TCOs in electronic applications. We also show the highest value of *σ* (300 K) of bulk BSO single crystals and thin films grown using different routes. Remarkably, the film grown using hybrid MBE possesses higher conductivity than those reported to-date both in the bulk BSO crystal and films, and is comparable to that of ITO, an industry standard for TCOs. We note that although high conductivity is achieved owing to high doping density and mobility, the ability of BSO to afford high conductivity without any phase segregation is very promising for fundamental study and application. For example, conductive BSO could be an ideal electrode material in oxide electronics providing not only electrode function but also transparency, which could be vital for applications such as solar cells or display technologies. In addition, high conductivity of BSO could also be important for fundamental study and applications at high frequencies. High-frequency measurements and devices require low resistance contacts, where epitaxially-grown highly conductive BSO can play a significant role. Moreover, high conductivity of BSO films can also be beneficial for the investigation of ultra-fast phase dynamics of multiferroics without potential issues of charging effect. The major advantage of using conductive BSO as opposed to metals is its structural compatibility with these materials. Finally, the ability of BSO to accommodate high density of electrons and mobility along with wide bandgap makes it a suitable material candidate for ultra-high density quantum wells for plasmonic and power electronics applications.

### Discussion

In summary, we have demonstrated the hybrid MBE approach for reproducible and controlled doping of epitaxial BSO on STO(001) with La. We establish a correlation between dislocation, 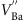, carrier density, mobility and metal-insulator transition in BSO. The combination of controlled doping, detailed electronic characterization, with modelling using the Boltzmann transport equations and an *ab initio* band structure calculations allow us to understand, tune and predict the transport behaviour and mobility limiting mechanisms in BSO. An important outcome of the study is that room temperature mobility in BSO films is limited by both defects, and electron-phonon scattering. The results place doped-BSO on par with highly successful III-Nitrides in terms of mobilities but with the added benefit of having high carrier concentrations for high-power oxide electronics operating at room temperature.

## Methods

### Growth and characterization of BaSnO_3_ films

Details of our hybrid MBE approach for stoichiometric BSO heteroepitaxy are discussed elsewhere^21^,^25^; a brief description will be provided here. BSO films were grown via hybrid molecular beam epitaxy (hybrid MBE). Hexamethylditin was used as the metal-organic chemical precursor for Sn. Ba and La were evaporated using effusion cells, and oxygen was supplied using an RF plasma source. The oxygen pressure and substrate temperature were fixed at 5 × 10^−6^ torr and 900 °C respectively for all growths. A 350-nm-thick Ta layer was deposited on the back of the substrates to improve heat transfer between the film and the substrate heater. Substrates were cleaned in oxygen plasma for 20 min before film growth ensuring no carbon contamination was present at the surface. Dopant density in the films was controlled by varying La-cell temperature (1060 °C≤*T*_La_ ≤1290 °C). Films were annealed after growth in oxygen using rapid thermal annealer at 800 °C for 2 min to ensure oxygen stoichiometry. All substrates after film growth and undoped films were confirmed to be insulating (*ρ*>10^5^ Ω cm). Electronic transport measurements were performed in a Quantum Design Physical Property Measurement System (DynaCool) using indium as ohmic contacts in a van der Pauw geometry. Thermopower measurements were performed with a home built system described elsewhere^40^.

### AMSET and DFT calculation details

The AMSET model employs materials parameters that were obtained from the experimental literature, as listed in Table 1. In this work, the primary purpose of the AMSET model is to explain the observed experimental phenomena, so it is appropriate to use experimental parameters even though the same values can be calculated *ab initio* with only minor deviations to the resulting mobility and Seebeck coefficient. For instance, deformation potential (*E*_D_) is calculated *ab initio*. We, however, emphasize that the choice of deformation potential in our calculations, even if an order of magnitude different than the value reported in Table 1 does not influence our interpretation of mobility data. It is noted that the mobility due to AC scattering, which uses *E*_D_ is not limiting at any temperature or concentration. The same holds true for the values of the elastic constants that were extracted from DFT generalized gradient approximation of Perdew, Burke and Ernzerhof (GGA-PBE) calculations^41^; these have essentially no effect on the overall mobility, as the mobility due to AC scattering is always orders of magnitude higher than the actual limiting mechanisms.

To calculate the electronic band structure of BSO, the unit cell was optimized using Kohn–Sham density functional theory (KS-DFT)^42^,^43^ as implemented in the Vienna *ab initio* Simulation Package^44^,^45^,^46^,^47^. We used the generalized gradient approximation of Perdew, Burke and Ernzerhof^48^ (GGA-PBE) to express the exchange-correlation potential and Projector Augmented Wave potentials to represent the wave functions. On geometry optimization, the lattice constant (0.419 nm) was increased by 1.76% compared to the experimental value (0.412 nm). This is a common behaviour when using a GGA-PBE functional, due to delocalization of electrons. We used a **k**-point mesh of 6 × 6 × 6 for self-consistent calculations, as adding more **k**-points results in <0.01 eV difference in total energy. The energy cutoff for the plane wave basis set was set to 520 eV in all calculations.

For electronic properties calculations, we chose the **k**-points closest to the conduction band minimum (CBM), which for BaSnO_3_ is at the Γ-point. An adaptive **k**-points mesh was used, which is denser close to the CBM. We used the AMSET code^35^ to calculate the mobility and the Seebeck coefficient, which uses the DFT band structures to solve the BTE. The explicit solution to BTE is particularly important for the inelastic LO-phonon scattering mechanism as it is the major limiting mechanism for the sample with *n*_3D_=4.06 × 10^20^ cm^−3^, for *T*>200 K (Fig. 4b). More information on the LO-scattering can be found elsewhere^35^,^39^. On the other hand, two other major elastic scattering mechanisms in BSO are ionized impurity (IMP) and charged dislocation scattering (DIS). We note that it is the threading dislocations in the active layer, which limit the mobility. Misfit dislocations on the other hand are pinned at the film/substrate interface making themselves electrically separated from the active layer where electronic transport is measured. We write the expressions used to model these scattering phenomena from refs 35, 39 to clarify how these scattering rates and necessary parameters are calculated. The IMP and DIS scattering rates, *ν*, are expressed via the following equations:


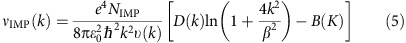



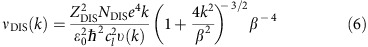



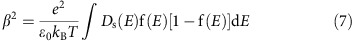


where *k* is the magnitude of the wave vector, *E* is the energy, *β* is the inverse screening length due to charge defects, *ɛ*_0_ is the static dielectric constant, *e* is the charge of an electron, *c*_l_ is the lattice parameter of BSO, *υ*(*k*) is the group velocity of electron which is directly derived from the DFT band structure, *Z*_DIS_ is the charge state of dislocations, *B*(*K*) and *D*(*K*) are parameters describing the non-parabolicity of the conduction band, which are directly derived from the DFT band structure, *D*_s_ is the density of states and *k*_B_ is the Boltzmann constant. We further note that to calculate the mobility values, *Z*_D_*Z*_DIS_*N*_DIS_ is used as a fitting parameter instead of *N*_DIS_ as the charge states of the donor (defined as charged dopants and other donor-like defects to compensate for charged dislocations) and dislocations are not known. It is interesting to note that the screening lengths are the same in Fig. 4b,c both of which have very high concentrations indicating a highly degenerate semiconductor or essentially a metal. In fact, the calculated Fermi level for Fig. 4a is 0.15 eV inside the conduction band (above the CBM) while that is 0.94 and 1.03 eV for samples with higher *n*_3D_. At such high concentrations, the departure from uniformity of the free-electron concentration in presence of a potential surrounding an impurity, *δn*, becomes insensitive to the concentration itself. *δn* is directly related to charge screening. See ref. 38 for more details.

### Data availability

The data that support the main findings of this study are available from the corresponding authors on request.

## Additional information

**How to cite this article:** Prakash, A. *et al*. Wide bandgap BaSnO_3_ films with room temperature conductivity exceeding 10^4^ S cm^−1^. *Nat. Commun.* **8,** 15167 doi: 10.1038/ncomms15167 (2017).

**Publisher's note:** Springer Nature remains neutral with regard to jurisdictional claims in published maps and institutional affiliations.

## Supplementary Material

Supplementary Information Supplementary Figures and Supplementary Reference

## Figures and Tables

**Table 1:** Input parameters to our AMSET model using *ab initio* calculations and using experimental values.

**Model parameters**	***Ab initio***	**Experiments**^56^
Polar optical phonon frequency, *ω*_PO_ (THz)	20.36	**12.23**
Low-frequency dielectric constant, *ɛ*_0_	5.2	**17±2**
High-frequency dielectric constant, *ɛ*_∞_	3.4	**3.3**
Deformation potential, *E*_D_ (eV)	**6.19**	–

The bold numbers indicate parameters that are used in our calculations.
