# Supplementary material for: Wide bandgap BaSnO3 films with room temperature conductivity exceeding 104 S cm−1
Source: Nat Commun. 2017 May 5;8:15167. doi: 10.1038/ncomms15167 (PMC5424175; doi:10.1038/ncomms15167)
Supplement: Supplementary Information — Supplementary Figures and Supplementary Reference [file ncomms15167-s1.pdf]

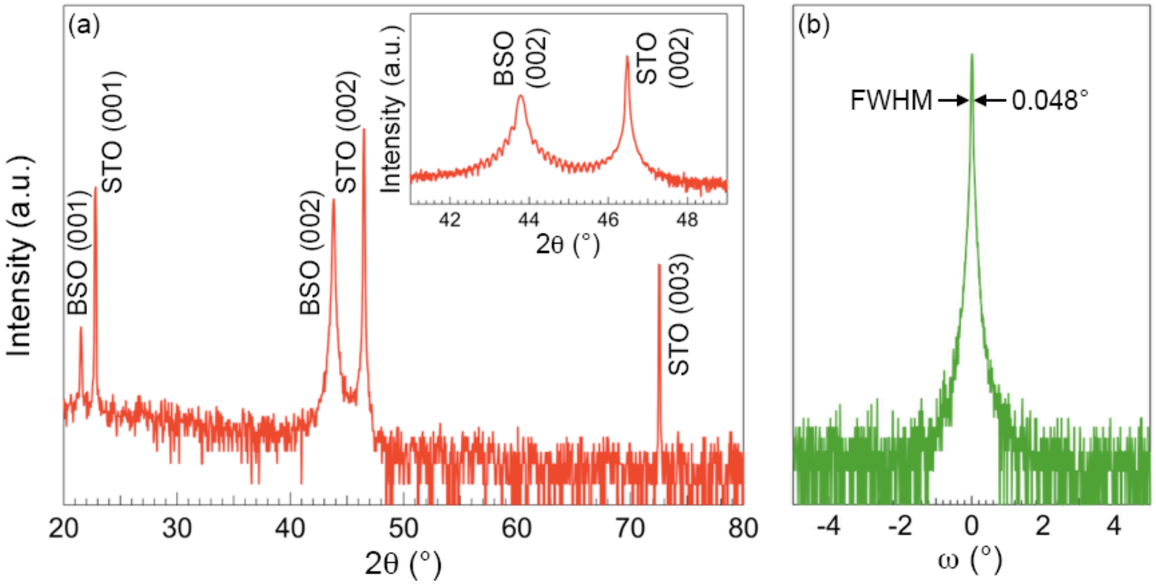

Supplementary Figure 1: (a) Log (intensity) vs. 2θ wide-angle X-ray coupled scan of a 62 nm $BaSnO_3$ film on $SrTiO_3$ (001) substrate indicating phase-pure and single crystalline film. The structure had a 31 nm of undoped $BaSnO_3$ buffer layer followed by 31 nm of La-doped $BaSnO_3$ active layer (n_{3D} at 300K = 2.53×10^{20} cm^{-3}). We are showing a representative XRD pattern for a thinner sample as opposed to thicker buffer layer sample to emphasize that these films are smooth also on short lateral length scales as evident by the presence of Kiessig's fringes. The Kiessig's fringes are smeared together for a thick film. The out-of-plane lattice parameter was calculated to be 4.131±0.001 Å, which is higher than the bulk value of 4.116 Å. The expanded lattice parameter is attributed to residual strain in the films. [See Ref. 1] (b) Rocking curve (ω scan) with respect to the (002) $BaSnO_3$ film diffraction peak indicating a FWHM of 0.048°. The substrate had an FWHM of 0.014°. Higher FWHM of the $BaSnO_3$ film is likely due to the strain relaxation.

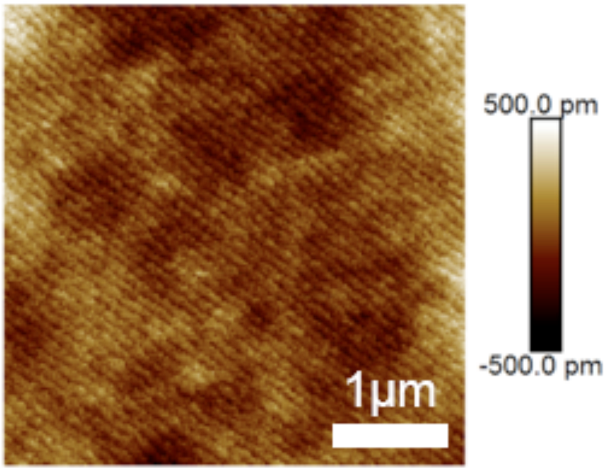

Supplementary Figure 2: Atomic force micrograph of a 26 nm La-doped $BaSnO_3$ film grown on $SrTiO_3$ substrate with a 26 nm buffer layer showing atomically smooth film with an r.m.s. roughness of 1.2 Å.

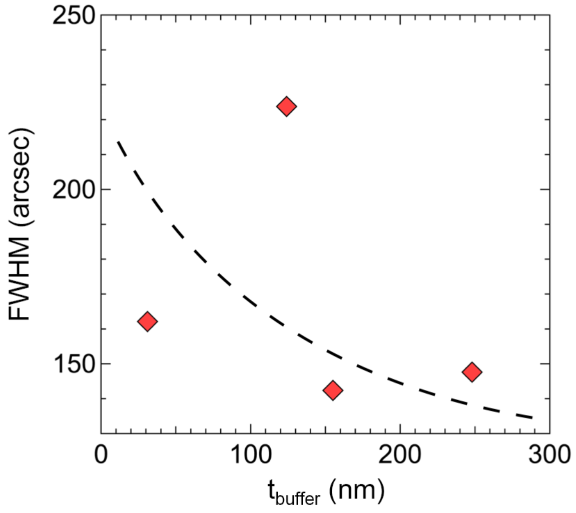

Supplementary Figure 3: Full-width at half maxima (FWHM) of the ω-scans for $BaSnO_3$ films with different buffer layer thickness (t_{buffer}). The plot shows a gradual decrease in the dislocation density (N_{DIS}) in these samples with increasing buffer layer thicknesses. Dashed line is a guide to the eye.

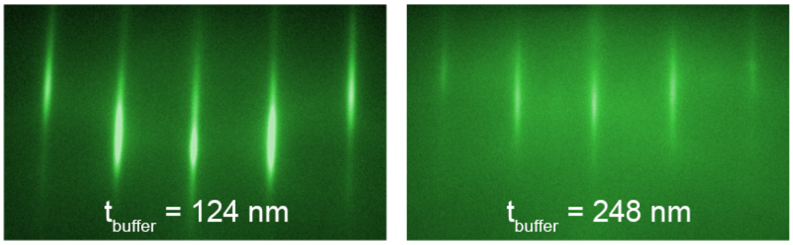

Supplementary Figure 4: RHEED patterns along [100] azimuth for samples grown with different buffer layer thickness. The pattern becomes more diffuse with increasing buffer layer thickness suggesting that the surface is becoming rough as t_{buffer} is increased.

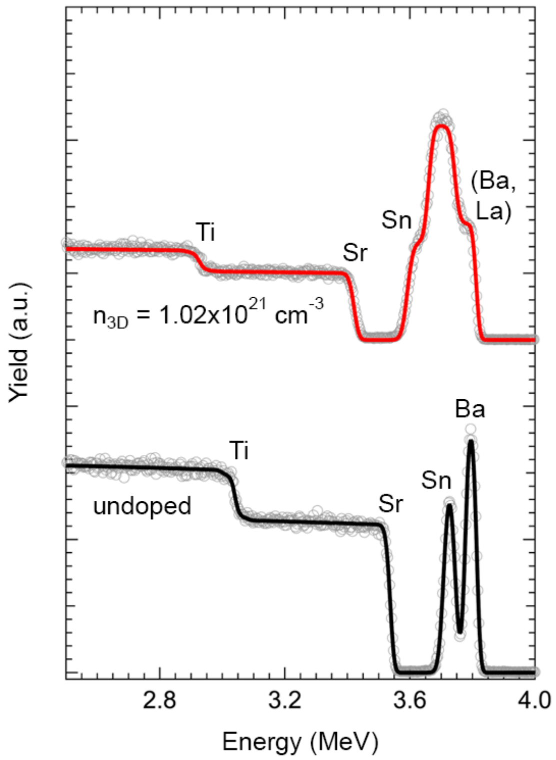

Supplementary Figure 5: Experimental (circular symbols) and simulated (solid lines) RBS spectra for a highly doped BSO film (124 nm doped + 124 nm undoped buffer on STO substrate) with $n_{3D} = 1.02\times10^{21}$ cm^{-3}, and an undoped BSO film (~56 nm) as a reference yielding Ba:Sn ratio of 1.002 ± 0.02 in undoped film, and 0.96 ± 0.02 in doped sample. Given these films are grown with identical growth conditions, film stoichiometry, i.e. atomic ratio of A- to B-site should be identical, i.e., Ba/Sn = 1 for undoped film, and (Ba+La)/Sn = 1 for doped film. We thus estimate La-dopant concentration in our doped sample to be about 8% ± 2% Note that RBS represents data from both 124 nm doped and 124 undoped buffer layer in the doped sample, which is accounted for in the estimation of La-dopant concentration. This value of La-concentration is in reasonable agreement with the number of active carrier density expected based on Hall measurements, suggesting that the source of carrier in our doped film is La and not any other extrinsic/intrinsic defects.

Supplementary Reference:

[1] Prakash, A. *et al.* Hybrid molecular beam epitaxy for the growth of stoichiometric $BaSnO_3$. *J. Vac. Sci. Technol. A* **33**, 060608 (2015).
